# OxyR senses sulfane sulfur and activates the genes for its removal in *Escherichia coli*

**DOI:** 10.1016/j.redox.2019.101293

**Published:** 2019-08-08

**Authors:** Ningke Hou, Zhenzhen Yan, Kaili Fan, Huanjie Li, Rui Zhao, Yongzhen Xia, Luying Xun, Huaiwei Liu

**Affiliations:** a State Key Laboratory of Microbial Technology, Shandong University, Qingdao, 266237, People's Republic of China; b School of Molecular Biosciences, Washington State University, Pullman, WA, 99164-7520, USA

**Keywords:** OxyR, Sulfane sulfur, *Escherichia coli*, Thioredoxin, Glutaredoxin

## Abstract

Sulfane sulfur species including hydrogen polysulfide and organic persulfide are newly recognized normal cellular components, and they participate in signaling and protect cells from oxidative stress. Their production has been extensively studied, but their removal is less characterized. Herein, we showed that sulfane sulfur at high levels was toxic to *Escherichia coli* under both anaerobic and aerobic conditions. OxyR, a well-known regulator against H_2_O_2_, also sensed sulfane sulfur, as revealed via mutational analysis, constructed gene circuits, and *in vitro* gene expression. Hydrogen polysulfide modified OxyR at Cys199 to form a persulfide OxyR C199-SSH, and the modified OxyR activated the expression of thioredoxin 2 and glutaredoxin 1. The two enzymes are known to reduce sulfane sulfur to hydrogen sulfide. Bioinformatics analysis indicated that OxyR homologs are widely present in bacteria, including obligate anaerobic bacteria. Thus, the OxyR sensing of sulfane sulfur may represent a preserved mechanism for bacteria to deal with sulfane sulfur stress.

## Highlights

• OxyR also senses sulfane sulfur stress and activates the genes for its removal.
• OxyR senses hydrogen polysulfide via persulfidation of OxyR at Cys^199^.
• OxyR responds to sulfane sulfur stress under both aerobic and anaerobic conditions.
• OxyR is widely distributed in bacterial genomes, including anaerobic bacteria.

## Graphical abstract

**Figure:**
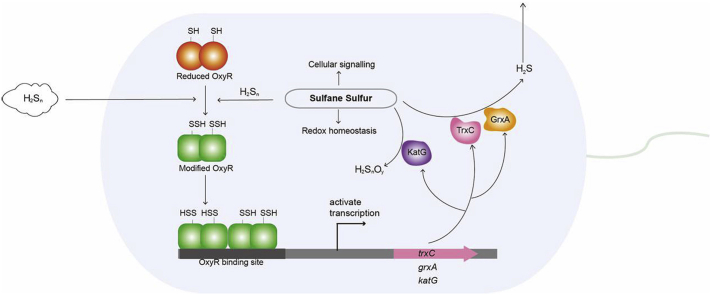

## 1 Introduction

Hydrogen sulfide (H_2_S) has been proposed as a gasotransmitter because it is involved in many physiological and pathological processes in animals and plants, such as ageing [1], neuromodulation [2], cancer cell proliferation, metabolic reprogramming [3,4], and stomatal closure in plant [5]. The mechanism of H_2_S signaling is often via protein persulfidation. H_2_S cannot directly react with protein thiols, but sulfane sulfur, its oxidation product, readily reacts with thiols to generate persulfides [6,7].

Sulfane sulfur species include hydrogen polysulfide (H_2_S_n_, n ≥ 2), organic polysulfide (RSS_n_H, RSS_n_R, n ≥ 2), and organic persulfide (RSSH), which can be produced from H_2_S oxidation or from the metabolism of cysteine and cystine. The endogenous H_2_S_n_ was initially discovered in rat brain [8]. Now, sulfane sulfur are considered as normal cellular components in both prokaryotic and eukaryotic cells [9,10]. Sulfane sulfur possesses both nucleophilic and electrophilic characteristics, while thiol (cysteine, GSH, etc.) is nucleophilic [11,12]. As nucleophiles, sulfane sulfur species are better reductants than thiols [13]; as electrophiles, they can transfer electrophilic sulfane sulfur (S^0^) to protein thiols to generate protein-SSH, affecting certain protein functions and protecting protein thiols from irreversible oxidation [14,15]. Owing to the dual-reactivities, sulfane sulfur is involved in many cellular processes, such as redox homeostasis, virulence in pathogenic bacteria, and biogenesis of mitochondria [16,17]. Sulfane sulfur also functions as antioxidants inside cells [18,19].

Albeit the good roles, sulfane sulfur may be toxic at high concentrations. Indeed, elemental sulfur has been used as an antimicrobial agent for ages, and its efficiency is likely impaired by its low solubility [20]. Advances in the synthesis of sulfur nanoparticles have significantly increased the antimicrobial efficiency of elemental sulfur [21]. Elemental sulfur is often used as a fungicide. Although its toxicity mechanism is unclear, a recent study suggested that sulfur is transported into the cell in the form of H_2_S_n_ [22], inducing protein persulfidation as a possible toxic mechanism [23]. Fungi may use glutathione to reduce polysulfide to H_2_S as a detoxification mechanism [22,24]. Organosulfur compounds can be used to treat antibiotic-resistant bacteria, and they are converted to H_2_S_n_ for the toxicity [25]. Both bacteria and fungi display reduced viability being exposed to sulfane sulfur in excess [22,25]. Therefore, intracellular sulfane sulfur is likely maintained within a range for microorganisms under normal conditions.

Multiple pathways for sulfane sulfur generation have been discovered. 3-Mercaptopyruvate sulfurtransferase and cysteinyl-tRNA synthetase produce sulfane sulfur from cysteine [8,26,27]. Cystathionine β-synthase and cystathionine γ-lyase can produce H_2_S from cysteine and sulfane sulfur from cystine [13]. Since cellular cystine concentration is very low, these enzymes are likely to generate H_2_S instead of sulfane sulfur [10]. Sulfide:quinone oxidoreductase and superoxide dismutase produce sulfane sulfur from H_2_S [28,29]. Most microorganisms possess several of these pathways. *Escherichia coli* contains 3-mercaptopyruvate sulfurtransferase, cysteinyl-tRNA synthetase, and superoxide dismutase that may generate cellular sulfane sulfur.

Microorganisms may use several mechanisms to remove or reduce sulfane sulfur inside cells. Aerobic microorganisms may apply persulfide dioxygenase to remove excessive sulfane sulfur [30], and the persulfide dioxygenase expression can be induced by sulfane sulfur via sulfane sulfur-sensing transcription factors [[31], [32], [33]]. Another possibility is that sulfane sulfur is reduced by glutathione (GSH) or glutaredoxin or thioredoxin to H_2_S [34,35], which is released out of cells [36], as observed in anaerobically cultured fungi [22,37]. A recent report that two thioredoxin-like proteins catalyze the reduction of protein persulfidation in *Staphylococcus aureus* also support that thioredoxin participates in the reduction of cellular sulfane sulfur to H_2_S [17]. However, it is unclear whether sulfane sulfur induces the expression of glutaredoxin and thioredoxin.

*E. coli*, a common intestinal bacterium, contains three thioredoxins and four glutaredoxins. The expression of TrxA, GrxB, and GrxD is regulated by guanosine 3′,5′-tetraphosphate, and the expression of GrxC is regulated by cAMP, both of which are nutrient-dependent messengers [38,39]. These four enzymes are highly abundant in *E. coli*; together they account for more than 1% of total protein [[40], [41], [42]]. The expression of GrxA, TrxC, and KatG (catalase) is regulated by OxyR upon exposure to H_2_O_2_. These proteins are much less than other thioredoxins and glutaredoxins in *E. coli* in the absence of oxidative stress [[41], [42], [43]].

OxyR was initially identified as a regulator responding to reactive oxygen species (ROS) [44,45]. ROS triggers the formation of a disulfide bond between Cys^199^ and Cys^208^ or oxidizes Cys^199^ to C199-SOH, but the exact mechanism is still in debate [[46], [47], [48]]. Herein, we showed that sulfane sulfur modified OxyR at Cys^199^ to form a protein persulfide that in turn activates the expression of thioredoxin, glutaredoxin, and catalase in *E. coli*, and the induced enzymes reduced cellular sulfane sulfur to H_2_S.

## 2 Materials and methods

### 2.1 Strains, plasmids, and chemicals

All strains and plasmids used in this study are listed in Table S2. Deletion of *oxyR* was performed following a reported method [49]. *E. coli* strains were grown in Lysogeny broth (LB) medium. Antibiotics (50 μg/ml) were added when required. SSP4 (3′,6′-Di(*O*-thiosalicyl)fluorecein) was purchased from DOJINDO MOLECULAR TECHNOLOGIES. H_2_S_n_ was prepared by following Kamyshny & Alexey's method [50]. Briefly, 13 mg of sulfur powder and 70 mg of sodium sulfide were added to 5 ml of anoxic distilled water under argon gas. The pH was adjusted to 9.3 with 6 M HCl. The obtained product contained a mixture of H_2_S_n_, where n varies from 2 to 8 [51], but at low concentration and neutral pH, H_2_S_2_ is dominant [29].

### 2.2 Cellular sulfane sulfur analysis

SSP4 probe was used for cellular sulfane sulfur analysis. *E. coli* cells (1 ml) were taken out from the culture at specific time points and diluted to OD_600nm_ = 1, washed, and resuspended in HEPES buffer (50 mM, pH 7.4); then 10 μM SSP4 and 0.5 mM CTAB were added. After an incubation at 37 °C for 15 min in the dark with gently shaking (125 rpm), reagents were washed off with HEPES buffer (50 mM, pH 7.4). Reacted-cells were subjected to flow cytometry (FACS) analysis by using BD Accuri™ C5. For each sample, >10,000 cells were analyzed in FL1-A channel. The average fluorescent intensity was used to estimate cellular sulfane sulfur of sampled cells.

The CstR-based reporting system was used for real-time analysis. *cstR* gene was chemically synthesized by Genewiz (Shanghai) company and expressed with *P*_*lacI*_ promoter in pTrcHis2A plasmid, where the *trc* promoter was replaced by the CstR cognate promoter, and a *mkate* gene (with a C-terminus degradation tag *ssrA*) was put after it (Table S2, entry 22). For *trxA*, *trxB*, *grxB*, *grxC*, or *grxD* overexpression experiment, the gene was introduced after *mkate* (Table S2, entries 23–27). *E. coli* strains containing reporting plasmids were culture in LB medium at 37 °C with shaking (220 rpm). Fluorescence was analyzed by FACS (FL3-A channel, >10,000 cells).

We used a reported method to quantitate the concentration of cellular sulfane sulfur [52]. Briefly, *E. coli* cells were harvested and resuspended (OD_600nm_ = 10) in 1 M NaOH, 0.1% SDS and 0.3 M ascorbic acid to lysis cells and reduce cellular sulfane sulfur to sulfide. Then zinc acetate was added to recover the released sulfide as ZnS precipitate. The precipitate was collected by centrifugation and washed with distilled water. The ZnS precipitate were resuspended in 1 mL distilled water and mix with 100 μL methylene blue reagent (30 mM FeCl_3_; 20 mM N*,N*-dimethyl-*p*-phenylenediamine; 7.2 M HCl ) to detect S^2−^ released from ZnS at 670 nm. Sulfane sulfur in the solution was converted to cellular sulfane sulfur by using a reported conversion factor: one mL of cells at OD_600nm_ of one was converted to one μL of cellular volume (https://bionumbers.hms.harvard.edu/search.aspx).

### 2.3 H_2_S production analysis

Production of H_2_S was determined by using a previously reported method [53]. Briefly, H_2_S was derivatized with mBBr then analyzed by HPLC (LC-20A, Shimadzu) equipped with a fluorescence detector (RF-10AXL, Shimadzu). A C18 reverse phase HPLC column (VP-ODS, 150 × 4 mm, Shimadzu) was pre-equilibrated with 80% Solvent A (10% methanol and 0.25% acetic acid) and 20% Solvent B (90% methanol and 0.25% acetic acid). The column was eluted with the following gradients of Solvent B: 20% from 0 to 10 min; 20%–40% from 10 to 25 min; 40%–90% from 25 to 30 min; 90%–100% from 30 to 32 min; 100% from 32 to 35 min; 100 to 20% from 35 to 37 min; and 20% from 37 to 40 min. The flow rate was 0.75 ml/min. For detection, the excitation wavelength was set to 340 nm and emission wavelength was set to 450 nm.

### 2.4 H_2_S_n_ inhibition and induction tests

For growth inhibition test, middle-log phased *E. coli* cells (OD_600nm_ = 0.8) were diluted and dripped in freshly prepared LB agar medium containing 0 or 100 μM H_2_S_n_ and incubated in 37 °C under aerobic conditions. For anaerobic conditions, the anaerobic LB agar plates were prepared in an anaerobic glove box and the dilution and drip of *E. coli* cells also performed in an anaerobic glove box, then incubated in an anaerobic incubator at 37 °C for 24 h. For promoter induction test, a *mkate* gene was put after *trxC*, *grxA*, or *katG* native promoter in pTrchis2A plasmid (Table S2, entries 17–19). The *oxyR* or its mutant gene was expressed under the *P*_*lacI*_ promoter in the same plasmid (Table S2, entries 5–16) for complementary experiments. The obtained plasmids were transformed into *wt* and *ΔoxyR* strains. Early log-phased *E. coli* cells (OD_600nm_ = 0.5, in liquid LB) were incubated with 600 μM H_2_S_n_ for 2 h. Cells were harvested and washed with HEPES buffer (50 mM, pH 7.4), then subjected to FACS analysis (FL3-A channel, >10,000 cells).

### 2.5 Real-time quantitative reverse transcription PCR (RT-qPCR)

RNA sample was prepared by using the TRIzol™ RNA Purification Kit (12183555,Invitrogen). Total cDNA was synthesized using the All-In-One RT Master Mix (ABM). For RT-qPCR, strains were grown in anaerobic LB medium until OD_600nm_ reached 0.4, and then 200 μM H_2_S_n_ were added into anaerobic bottle. After 60 min, cells were collected by centrifugation and RNA was extracted. RT-qPCR was performed by using the Bestar SybrGreen qPCR Mastermix (DBI) and LightCycler 480II (Roche). For calculation the relative expression levels of tested genes, GAPDH gene expression was used as the internal standard.

### 2.6 Protein purification and reaction with DTT or H_2_S_n_

The *oxyR* gene with a C-terminal His tag was ligated into pET30. Mutants of *oxyR* were constructed from this plasmid via site-directed mutagenesis [54]. The obtained plasmids were transformed into *E. coli* BL21 (DE3). For protein expression, *E. coli* cells were cultured in LB medium at 25 °C with shaking (150 rpm) until OD_600nm_ reacted 0.6–0.8, 0.4 mM isopropyl-β-d-thiogalactopyranoside (IPTG) was added, and cells were cultured for additional 16 h at 16 °C. Cells were then harvested and disrupted through a high pressure cracker SOCH-18 (STANSTED); protein was purified via the Ni-NTA resin (Invitrogen). Buffer exchange of the purified protein was performed by using PD-10 desalting column (GE Healthcare).

Reactions were performed in an anaerobic glove box. 0.6 mg/ml protein was mixed with 200 mM DTT in a pH 8.0 buffer (50 mM NaH_2_PO_4_, 300 mM NaCl). After 1-h incubation at RT, the protein was dialyzed against 0.5 M KCl until the dialysis buffer was free of DTNB-titratable SH group. For H_2_S_n_ or H_2_O_2_ reaction, the mole ratio of reduced OxyR to H_2_S_n_ or H_2_O_2_ was 1:10. After incubating the mixture for 30 min at RT, unreacted H_2_S_n_ or H_2_O_2_ was removed via dialysis. The reacted-proteins were sealed and taken out from the glove box to be used in further experiments.

### 2.7 LC-MS/MS analysis of OxyR

The H_2_S_n_-reacted OxyR (0.5 mg/ml) was mixed with iodoacetamide (IAM), and then digested with trypsin by following a previously reported protocol [32]. The Prominence nano-LC system (Shimadzu) equipped with a custom-made silica column (75 μm × 15 cm) packed with 3-μm Reprosil-Pur 120C18-AQ was used for the analysis. For the elution process, a 100 min gradient from 0% to 100% of solvent B (0.1% formic acid in 98% acetonitrile) at 300 nl/min was used; solvent A was 0.1% formic acid in 2% acetonitrile. The eluent was ionized and electrosprayed via LTQ-Orbitrap Velos Pro CID mass spectrometer (Thermo Scientific), which run in data-dependent acquisition mode with Xcalibur 2.2.0 software (Thermo Scientific). Full-scan MS spectra (from 400 to 1800 *m*/*z*) were detected in the Orbitrap with a resolution of 60,000 at 400 *m*/*z*.

### 2.8 In vitro transcription-translation analysis

*In vitro* translation-transcription reactions were performed using the Purexpress *In Vitro* Protein Synthesis system (NEB #E6800). The reaction solution was prepared in the following order: 10 μL solution A (NEB #E6800), 7.5 μL solution B (NEB #E6800), 2 μL E*. coli* RNA polymerase (NEB #M0551), 1 μL RNase inhibitor, 500 ng reduced, H_2_S_n_-treated or H_2_O_2_-treated protein, 200 ng DNA fragment containing *P*_*trxC*_*-mKate*, and RNase free water. The total volume was 25 μL. The solution was incubated at 37 °C for 3 h. After reaction, the translated mKate was diluted four times with distilled water, and assayed by using the Synergy H1 microplate reader. The excitation wavelength was set to 588 nm, and the emission wavelength was set to 633 nm. The fluorescence intensity from reduced OxyR was used as standard; fluorescence intensities from other groups were divided by the standard to calculate the relative expression levels.

### 2.9 Transcriptomic analysis

*E. coli wt* strain was cultured in LB medium until OD_600nm_ reached 0.5, and 500 μM H_2_S_n_ or 500 μM H_2_O_2_ were added. After 20 min of treatment, cells were harvested and total RNA was extracted by using the TRIzol™ RNA Purification Kit (12183555,Invitrogen). RNA quality was assessed with the RNA Nano 6000 Assay Kit of the Agilent Bioanalyzer 2100 system (Agilent Technologies). rRNA was removed with the Ribo-Zero rRNA Removal Kit (MRZMB 126, Epicentre Biotechnologies). For cDNA library construction, first-strand cDNA was synthesized by using random hexamer primers from fragmentation of mRNA and second-strand cDNA was synthesized by using a dNTP mixture containing dUTP with DNA polymerase I and RNase H. After adenylation of the ends of blunt-ended DNA fragments, NEBNext index adaptor oligonucleotides were ligated to the cDNA fragments. The second-strand cDNA containing dUTP was digested with the USER enzyme. The first-strand DNA fragments with ligated adaptors on both ends were selectively enriched in a 10-cycle PCR reaction, purified (AMPure XP), and the library was quantified using the Agilent High Sensitivity DNA assay on the Agilent Bioanalyzer 2100 system. The library was sequencing on Illumina Hiseq 2500 platform. Sequencing was performed at Beijing Novogene Bioinformatics Technology Co., Ltd. The clean data were obtained from raw data by removing reads containing adapter, poly-N and low quality reads. The clean reads were aligned with the genome of *E. coli* BL21 by using Bowtie2-2.2.3. Gene expression was quantified as reads per kilobase of coding sequence per million reads (RPKM) algorithm. Genes with a p-value<0.05 found by DESeq and change fold>1.5 were considered as significantly differentially expressed. Gene Ontology (GO) and KEGG analyses were performed at NovoMagic platform provided by Beijing Novogene Bioinformatics Technology Co., Ltd.

### 2.10 Analysis of OxyR distribution in sequenced bacterial genomes

A microbial genomic protein sequence set from NCBI updated until November 11, 2017 was downloaded for OxyR search. The query sequences of OxyR were reported OxyR proteins [46,55,56] were used to search the database by using Srandalone BLASTP algorithm with conventional criteria (e-value ≤ 1e^−5^, coverage ≥ 45%, identity ≥ 30%) to obtain OxyR candidates from 8286 bacterial genomes. A conserved domain PBP2_OxyR and PRK11151 were used as standard features for further filtration of OxyR candidates. The candidates containing PBP2_OxyR or PRK11151 were identified as putative OxyR.

## 3 Results

### 3.1 The accumulation and reduction of endogenous sulfane sulfur in *E. coli*

**Fig. 1:**
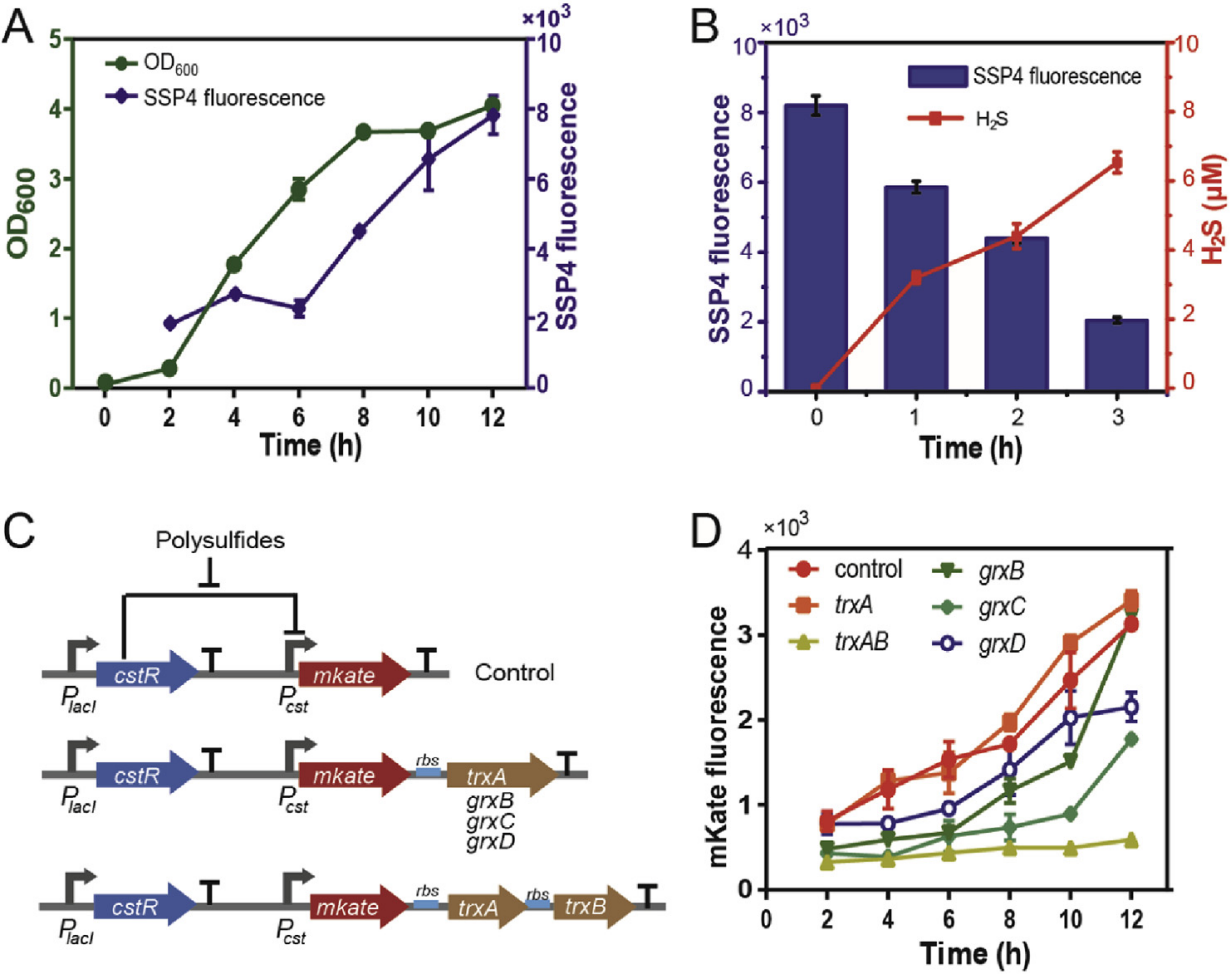
**Endogenous sulfane sulfur production and reduction in *E. coli*.** A *E. coli* accumulated sulfane sulfur during late-log and stationary phases. (n ≥ 3 for each group). B *E. coli* cells at stationary phase reduced intracellular sulfane sulfur to H_2_S after being transferred to fresh LB. (n ≥ 3 for each group). C CstR-based reporters for real-time monitoring intracellular sulfane sulfur. D Overexpression of thioredoxins and glutaredoxins decreased intracellular sulfane sulfur, as indicated by mKate fluorescence. (n ≥ 3 for each group). Data information: In (A, B and D), data are presented as mean ± SEM.

*E. coli* cells were cultured in LB medium and harvested at various incubation time. Cellular sulfane sulfur of the sampled cells was determined by using the sulfane sulfur sensitive probe SSP4. The cellular sulfane sulfur started to accumulate at mid-log phase and reached the maximum at early stationary phase (Fig. 1A). With the ascorbate reduction method [52], we determined cellular sulfane sulfur in cells harvested at 10 h as 381 ± 69 μM, similar to a previous report that the endogenous GSSH of mice brain tissue is around 150 μM [13]. When the stationary-phased cells were transferred into fresh medium (OD_600nm_ = 1), their intracellular sulfane sulfur decreased quickly with concomitant release of H_2_S (Fig. 1B). This phenomenon suggests that sulfane sulfur may be reduced to H_2_S by enzymes, such as thioredoxin and glutaredoxin [34,35].

### 3.2 Thioredoxin and glutaredoxin participate in the reduction of intracellular sulfane sulfur

To confirm the change of intracellular sulfane sulfur, we constructed a transcription factor (TF)-based reporting plasmid, which contains a sulfane sulfur-sensing TF (CstR) [33], its cognate promoter (*P*_*cst*_), and a red fluorescent protein (mKate, with a C-terminus degradation tag ssrA) (Fig. 1C). Using the reporting plasmid, the increase of intracellular sulfane sulfur in live cells (Fig. 1A) was reported as the mKate fluorescence (Fig. 1D). When GrxB, GrxC, or GrxD was co-transcribed with mKate under the control of CstR, their expression could partially decrease the sulfane sulfur accumulation as reflected by the decreased mKate fluorescence intensity (Fig. 1D). TrxA alone did not affect sulfane sulfur accumulation (Fig. 1D); however, the co-transcription of mKate with TrxA and TrxB (thioredoxin reductase) prevented the increase of sulfane sulfur during the log phase of growth (Fig. 1D). These results confirmed that thioredoxin and glutaredoxin also reduce sulfane sulfur *in vivo*.

The artificial operons with thioredoxin or glutaredoxin created negative feedback loops (Fig. 1C&D), maintaining the intracellular sulfane sulfur within a narrow range that was defined by the leaky strength of *Pcst* and the sensitivity of CstR as well as the reductase activity. Since OxyR is known to regulate similar enzymes, we speculated whether OxyR may function in a similar way as CstR in the artificial operons (Fig. 1C).

### 3.3 *E. coli* ΔoxyR is more sensitive to H_2_S_n_ than wt

**Fig. 2:**
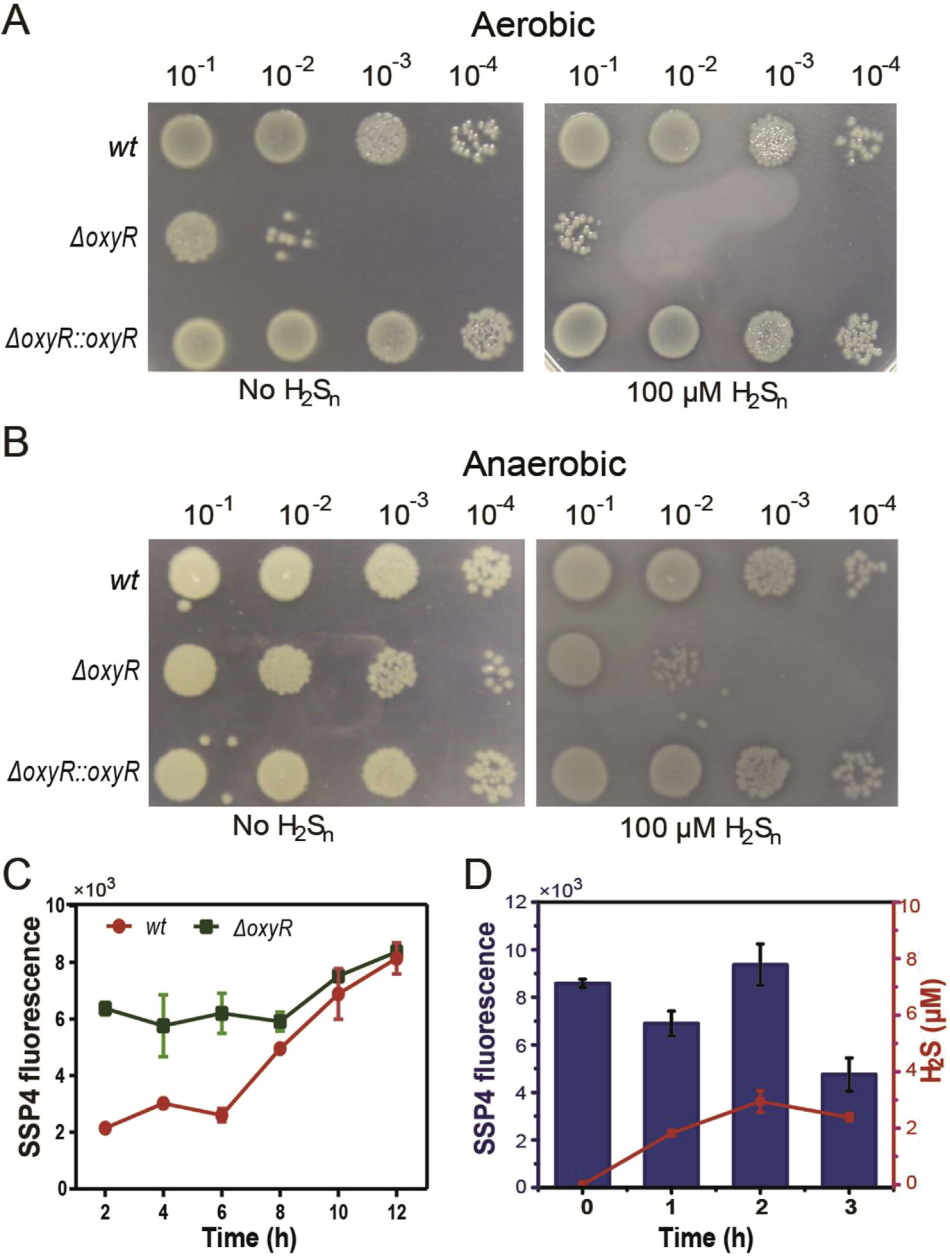
**OxyR affects RSS reduction in *E. coli*.** A, B The *oxyR* deletion made *E. coli* more sensitive to exogenous H_2_S_n_ stress under both aerobic and anaerobic conditions; the *oxyR* complementation restored the muntant's resistance to H_2_S_n_ stress. C *E. coli ΔoxyR* accumulated more endogenous sulfane sulfur than *E. coli wt* during growth in LB. (n ≥ 3 for each group). D After *E. coli* cells at stationary phase were transferred into fresh LB medium, *E. coli ΔoxyR* cells reduced endogenous sulfane sulfur to H_2_S more slowly than *E. coli wt* as shown in Fig. 1B. (n ≥ 3 for each group). Data information: In (C, D), data are presented as mean ± SEM.

We deleted *oxyR* gene in *E. coli* and observed that the mutant became more sensitive to exogenously added H_2_S_n_ under both aerobic and anaerobic conditions (Fig. 2A and B). In LB medium without added H_2_S_n_, *E. coli ΔoxyR* displayed similar growth as *wt*; however, on LB agar plates the deletion clearly showed dexterous effects on the growth for *E. coli ΔoxyR* under aerobic conditions. After complementing *oxyR* into *E. coli ΔoxyR*, the strain regained the tolerance to H_2_S_n_ (Fig. 2A and B). The results indicated that OxyR plays an important role in dealing with the exogenous H_2_S_n_ stress and the effects are more dramatic under anaerobic conditions. In addition, we noticed that *E. coli* showed higher H_2_S_n_ resistance under anaerobic condition compared with that of under aerobic condition, suggesting that oxidative stress may be involved under aerobic conditions.

*E. coli ΔoxyR* had higher intracellular sulfane sulfur than *wt* did at log-phase (Fig. 2C). When *E. coli ΔoxyR* cells at the stationary phase were transferred into fresh LB medium at OD_600nm_ of 1, the decrease of intracellular sulfane sulfur and the release of H_2_S were slower than that of the *wt* cells (Fig. 2D). The results suggested that OxyR regulates the production of thioredoxin and glutaredoxin that reduce sulfane sulfur to H_2_S.

### 3.4 OxyR regulates the expression of trxC, grxA and katG under both aerobic and anaerobic conditions

**Fig. 3:**
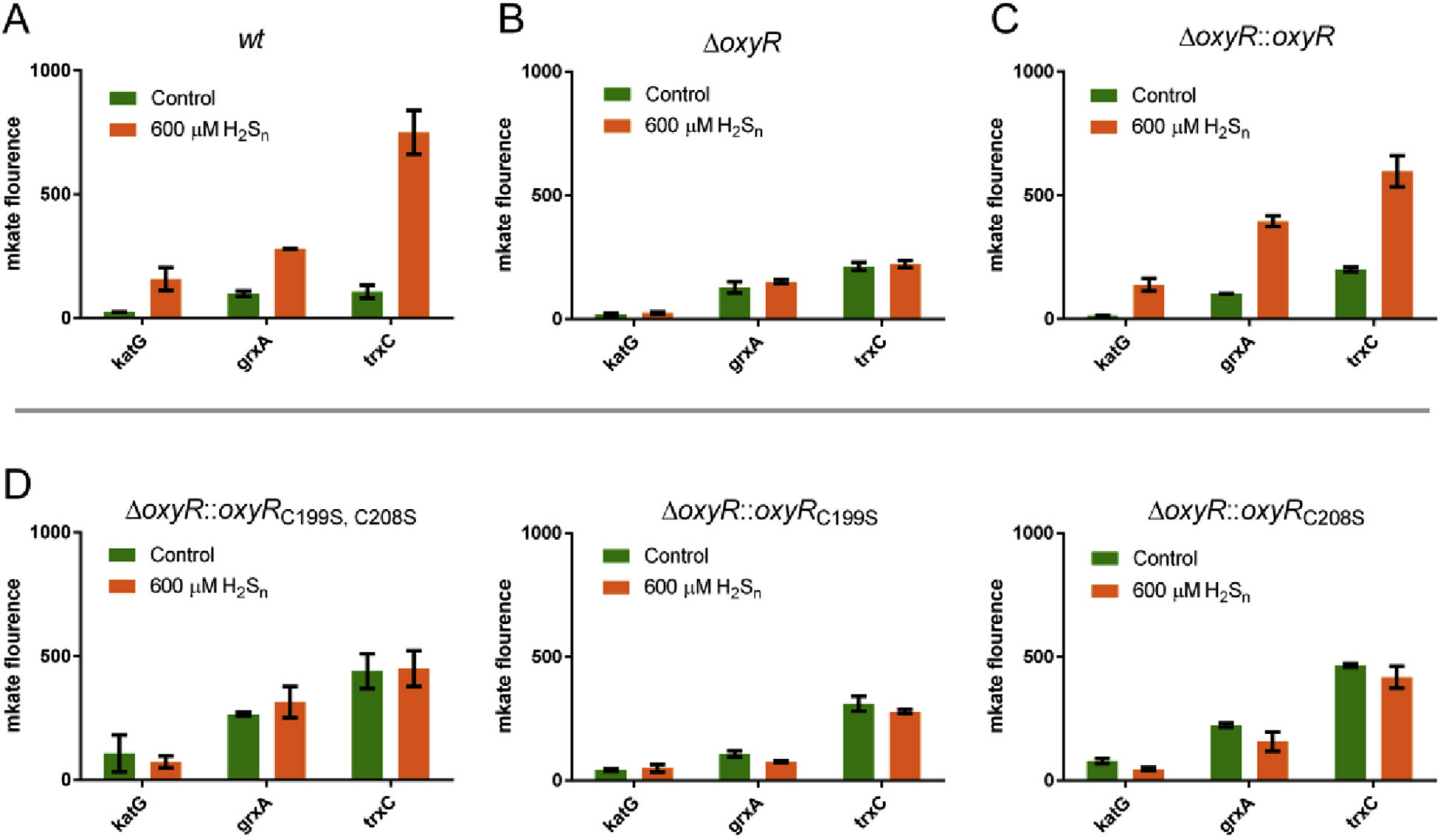
**H**_**2**_**S**_**n**_**upregulated expression of *katG*, *grxA*, and *trxC* via OxyR under aerobic conditions.** A H_2_S_n_ induced the expression of *katG*, *grxA*, and *trxC* in *E. coli wt*. (n ≥ 3 for each group). B The induction effect was lost in *E. coli ΔoxyR.* (n ≥ 3 for each group). C OxyR complementation recovered the induction effect. (n ≥ 3 for each group). D Cys199 and Cys208 single or double mutants lost the induction effect. (n ≥ 3 for each group). Data information: In (A–D), data are presented as mean ± SEM.

We constructed three reporting plasmids with an *mKate* gene under the control of the *trxC*, *grxA*, or *katG* promoter. These plasmids were transformed into *E. coli wt* and *ΔoxyR*, and the recombinant cells were tested under aerobic condition. In *wt*, all three promoters led to low *mKate* expression in the absence of H_2_S_n_, but resulted in obviously higher expression when H_2_S_n_ was added (Fig. 3A). In *E. coli ΔoxyR*, the three promoters led to constantly low expressions of *mKate* with or without added H_2_S_n_ (Fig. 3B). Complementation of *oxyR* restored the mutant's response to H_2_S_n_ (Fig. 3C). On the flipside, the introduction of plasmids overexpressing *trxC*, *grxA*, or *katG* in *E. coli ΔoxyR* decreased intracellular sulfane sulfur (Fig. S1).

Since the H_2_S_n_ solution contained sulfide, we tested if sulfide alone could induce the gene expression. Sulfide did not induce the expression of related genes in *wt* (Fig. S2 A), excluding the signal function of sulfide. When we used *E. coli* cells harboring a sulfide:quinone oxidoreductase of *C. pinatubonensis* JMP134, the added sulfide was oxidized to H_2_S_n_ [29], which induced the expression of *trxC*, *grxA* and *katG* (Fig. S2 B).

**Fig. 4:**
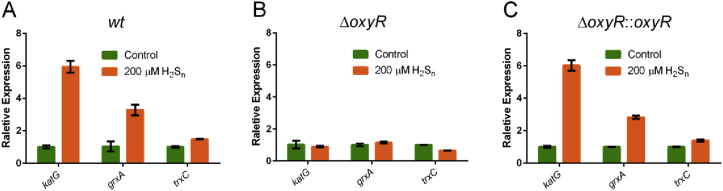
**H**_**2**_**S**_**n**_**upregulated expression of *katG*, *grxA*, and *trxC* via OxyR under anaerobic conditions**. A H_2_S_n_ induced the expression of *katG*, *grxA*, and *trxC* in *E. coli wt*. (n ≥ 3 for each group). B The induction effect was lost in *E. coli ΔoxyR.* (n ≥ 3 for each group). C OxyR complementation recovered the induction effect. (n ≥ 3 for each group). Data information: Data are presented as mean ± SEM.

We also tested the H_2_S_n_ induction under anaerobic conditions. Since mKate is not fluorescent under anaerobic condition, the gene expression was assayed by using qPCR. Similarly, *katG*, *grxA*, and *trxC* had higher expression in *wt* when 200 μM H_2_S_n_ were added (Fig. 4A), but not in *E. coli ΔoxyR* (Fig. 4B). Less H_2_S_n_ was required under anaerobic conditions than under aerobic conditions, likely due to the increased stability. After complementation, *E. coli ΔoxyR::oxyR* resumed response to H_2_S_n_ (Fig. 4C).

### 3.5 H_2_S_n_-treated OxyR activates the transcription of TrxC

**Fig. 5:**
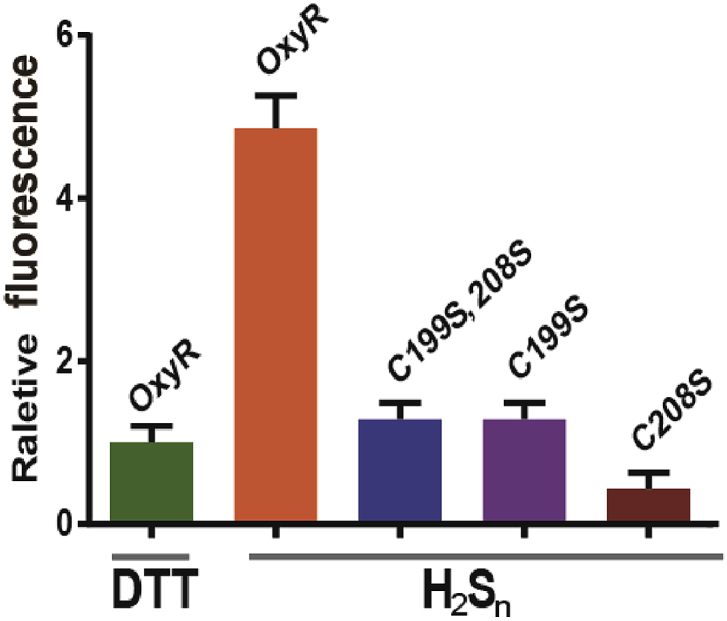
***In vitro* transcription-translation analysis of H**_**2**_**S**_**n**_**activation of OxyR and its mutants.** Purified OxyR and its mutants were treated with DTT to ensure their thiols were in the reduce form; The proteins were then treated with H_2_S_n_ to generate H_2_S_n_ modified protein. The *in vitro* transcription-translation system contained *P*_*trxC*_*-mKate* DNA fragment (200 ng) and DTT-reduced or H_2_S_n_-treated OxyR (500 ng), and the expressed mKate was analyzed with the fluorescence photometer Synergy H1. (n ≥ 3 for each group) Data information: Data are presented as mean ± SEM.

The activation of OxyR by H_2_S_n_ was tested with *in vitro* transcription-translation assays*.* The purified OxyR was treated with DTT to ensure that its thiols were in the reduced form, and the reduced OxyR was further treated with H_2_S_n_ to activate OxyR. The DTT-treated or H_2_S_n_-treated OxyR was used for *in vitro* transcription-translation of a DNA fragment containing the *trxC* promoter and *mKate* (*P*_*trxC*_*-mKate*). The DTT-treated OxyR resulted in low expression of *mKate*, while the H_2_S_n_-treated OxyR led to high expression of *mKate* (Fig. 5). These results indicated that H_2_S_n_ modifies OxyR, which enhances the expression from the *trxC* promoter.

### 3.6 Compare the induction effect of H_2_S_n_ with that of H_2_O_2_

**Fig. 6:**
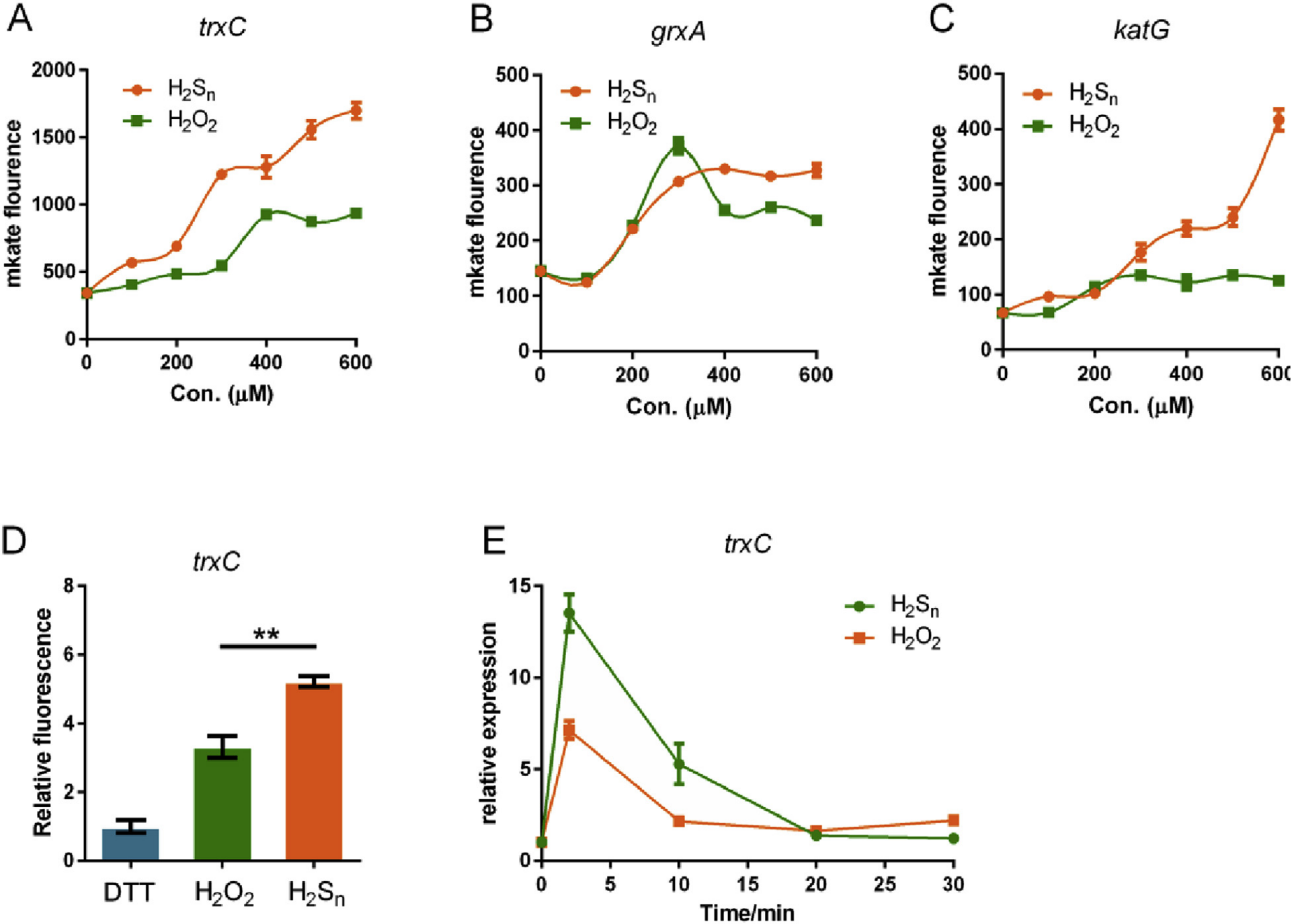
Comparison of the activation effect of H_2_S_n_ and H_2_O_2_. A-C H_2_S_n_ or H_2_O_2_ (100–600 μM) was used to treat *E. coli* wt strains containing reporter plasmids. (n ≥ 3 for each group) D Purified OxyR and its mutants were treated with DTT to ensure their thiols were in the reduce form; The proteins were then treated with H_2_S_n_ or H_2_O_2_ to generate H_2_S_n_- or H_2_O_2_-modified OxyR. The *in vitro* transcription-translation system contained *P*_*trxC*_*-mKate* DNA fragment (200 ng) and DTT-reduced, H_2_S_n_- or H_2_O_2_-treated OxyR (500 ng) and the expressed mKate was analyzed with the fluorescence photometer Synergy H1. (n ≥ 3 for each group) E H_2_S_n_ or H_2_O_2_ (400 μM) was used to treat *E. coli* wt. RT-qPCR was used to quantify the expression of *trxC*. (n ≥ 3 for each group).

We used 100–600 μM H_2_S_n_ or H_2_O_2_ to treat *E. coli* wt strains containing the reporting plasmids as mentioned in 3.4. At 100–200 μM level, H_2_S_n_ and H_2_O_2_ showed similar activation effects on *trxC*, *grxA* and *katG* promoters. However, at the dosage >400 μM level, H_2_S_n_ had obviously higher activation effects than H_2_O_2_ (Fig. 6A–C). For confirmation, we also compared their activation effects using *in vitro* transcription-translation with DTT-reduced, H_2_S_n_-treated, or H_2_O_2-_treated OxyR (500 ng). H_2_S_n_ also showed higher activation effect than H_2_O_2_ in *in vitro* transcription-translation of *mKate* (Fig. 6D).

In addition, we analyzed the expression induced by adding H_2_S_n_ or H_2_O_2_ to *E. coli* cells. The RT-qPCR results showed that once H_2_S_n_ (600 μM) was added, expression of *trxC* was rapidly increased in 2 min, indicating H_2_S_n_ quickly reacts with OxyR. However, the expression was significantly decreased at 10 min and finally dropped to untreated level at 20 min H_2_O_2_ (600 μM) addition showed the same trend, but with less expression of *trxC* (Fig. 6E).

### 3.7 H_2_S_n_-treatment causes the persulfidation of OxyR Cys^199^*in vitro*

OxyR contains six cysteine residues. Previous studies indicated that two of them (Cys^199^ and Cys^208^) are involved in ROS sensing [48]. We constructed an OxyR_4C→A_ mutant (except for Cys^199^ and Cys^208^, the other four cysteines were mutated to alanines) and expressed it in *ΔoxyR*. The mutant regulated *trxC*, *grxA*, and *katG* promoters essentially the same as the wild-type OxyR in the presence of H_2_S_n_. Whereas, OxyR_C199S_, OxyR_C208S_, and OxyR_C199S; C208S_ all lost the regulation function (Figs. 3D and 5). Together, these results indicated that the same as in ROS sensing, Cys^199^ and Cys^208^ are involved in H_2_S_n_ sensing.

**Fig. 7:**
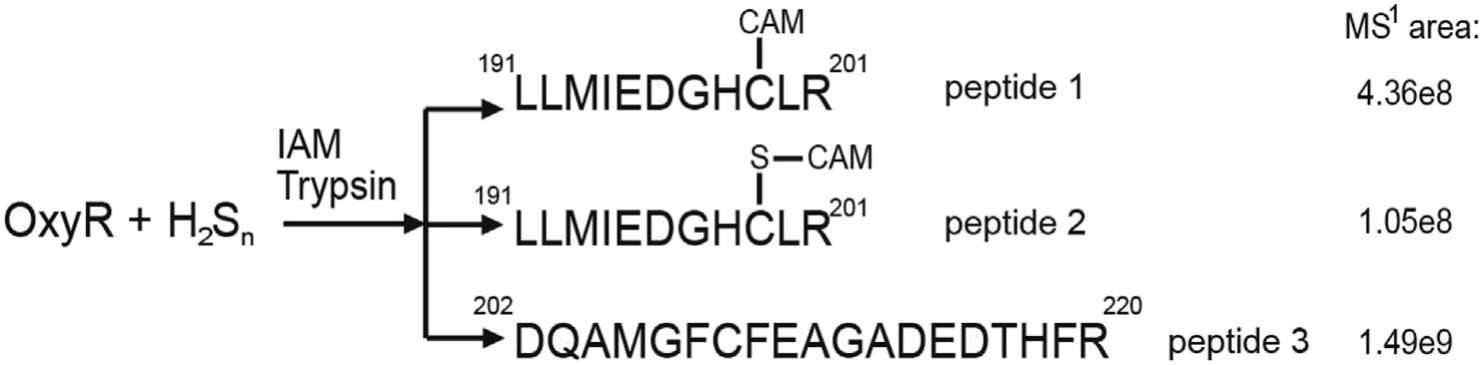
**LTQ-Orbitrap tandem mass analysis of H**_**2**_**S**_**n**_**-reacted OxyR.** Data information: MS data of the peptides are provided in Fig. S3∼5.

To find out the molecular mechanism on how OxyR senses H_2_S_n_, mass spectrometry analysis was performed to analyze the H_2_S_n_-treated OxyR. A short peptide (MW: 1356.67) containing Cys^199^ but not Cys^208^ was identified (peptide 1, Fig. 7 and Fig. S3) and about 20% of it contained Cys^199^-SSH (MW: 1388.64) (peptide 2, Fig. 7 and Fig. S4), according to the peak area in MS^1^ spectrogram. A peptide containing Cys^208^ was also found, but the Cys^208^ was not modified by iodoacetamide (IAM) (MW: 2144.87) (peptide 3, Fig. 6 and Fig. S5), which is consistent with a previous report that Cys^208^ is buried in the protein and is not accessible to IAM [47]. No peptide containing both Cys^199^ and Cys^208^ was detected. The *in vitro* experiments indicated that H_2_S_n_ reacts with Cys^199^ of OxyR, generating Cys^199^ persulfidation with no detectable disulfide or –S_n_– (n ≥ 3) bond between Cys^199^ and Cys^208^.

### 3.8 Global transcriptome analysis of H_2_S_n_-stressed and H_2_O_2_-stressed *E. coli*

**Fig. 8:**
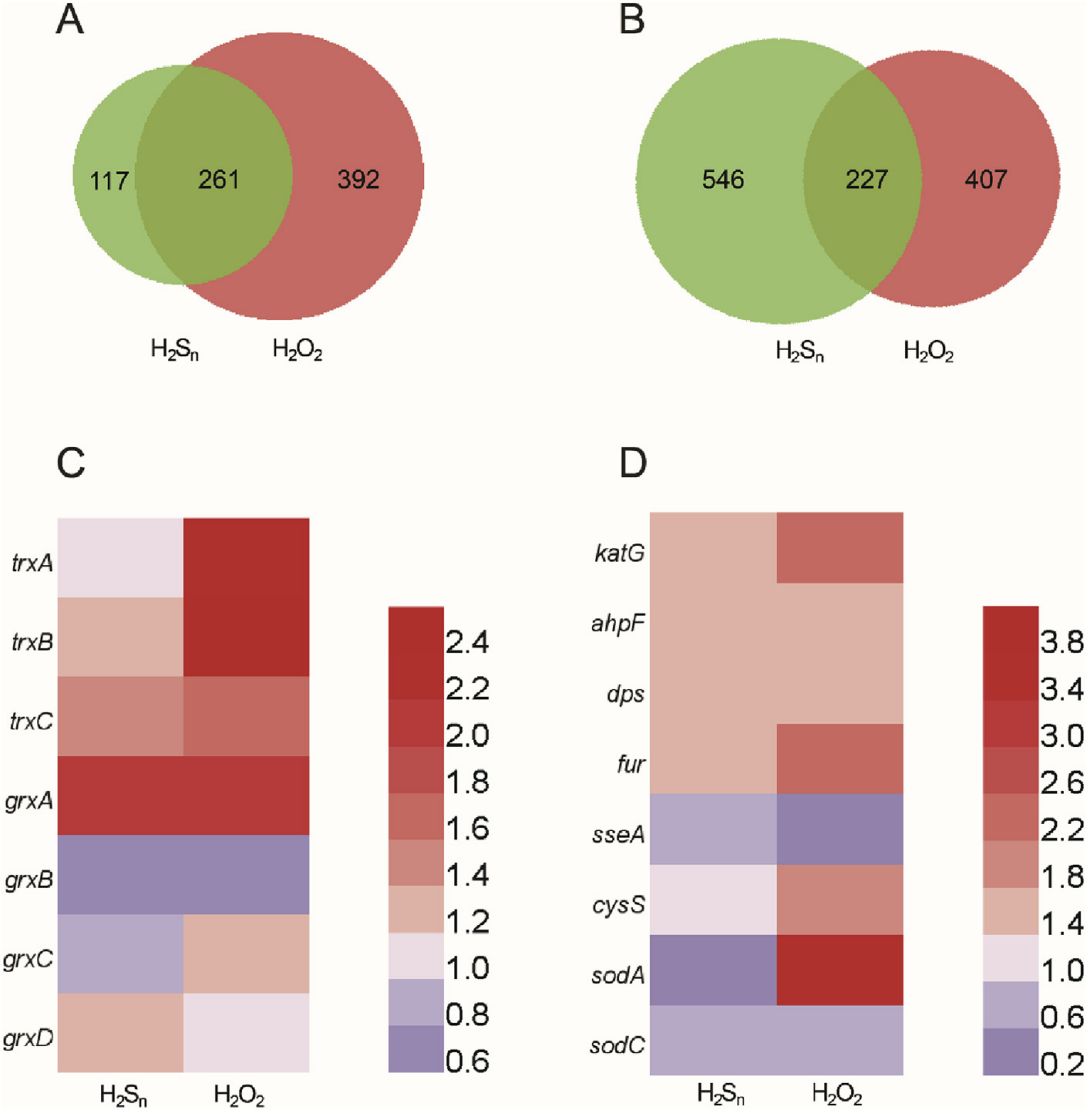
**Transcriptome analysis of H**_**2**_**S**_**n**_**- or H**_**2**_**O**_**2**_**-stressed *E. coli*.** A Numbers of transcriptionally upregulated genes under H_2_S_n_ and H_2_O_2_ stresses. B Numbers of transcriptionally downregulated genes under H_2_S_n_ and H_2_O_2_ stresses. C, D Transcriptional changes of genes related to sulfane sulfur and oxidative stress.

The effects of H_2_S_n_ stress and H_2_O_2_ stress on gene expression in *E. coli* were tested. H_2_O_2_ had more upregulated genes (Fig. 8A), while H_2_S_n_ had more down regulated genes (Fig. 8B). Both had some overlaps. At the global level, there were similarities and differences. Gene ontology (GO) analysis indicated the cellular processes affected by them were different. For instance, H_2_S_n_ stress upregulated more genes pertaining to cellular components, e.g., cell part (GO:0044464) and macromolecular complex (GO:0032991), and downregulated more genes pertaining to molecular transducer activity (GO:0060089) and signal transducer activity (GO:0004871); whereas H_2_O_2_ stress upregulated more genes pertaining to ribonucleotide binding (GO:0032553) and carbohydrate derivative binding (GO:0097367), and downregulated no gene pertaining to cellular components (Fig. S6 and Fig. S7). The TCA cycle is upregulated by H_2_S_n_ stress but downregulated by H_2_O_2_ stress; biosynthesis of secondary metabolites (i.e. serine hydroxymethyltransferase, beta-gulcosidase, 3-deoxy-7-phosphoheptulonate synthase, etc.) is downregulated by H_2_S_n_ stress but not affected by H_2_O_2_ stress (Fig. S8 and Fig. S9).

For the genes related to oxidative stress or sulfane sulfur, the effects on the expression of *grxA*, *grxB*, *ahpF*, *dps*, *sodC* were the same (Fig. 8C&D). For *trxB*, *trxC*, and *katG*, the degrees of upregulation were different (Fig. 8C&D). The expression of *sseA* encoding 3-mercaptopyruvate sulfurtransferase was down regulated by H_2_S_n_ and H_2_O_2_. The expression of *cysS* encoding cysteinyl-tRNA synthetase was not affected by H_2_S_n_, but upregulated by H_2_O_2_. For *grxC* and *sodA*, the effects of H_2_S_n_ and H_2_O_2_ were opposite (Fig. 8D). For *fur*, its expression was upregulated in both H_2_S_n_-and H_2_O_2_-treated cells. Fur is the repressor of iron importer and its upregulation can decrease cellular concentration of ferrous iron [57], minimizing hydroxyl radical production via the Fenton reaction when *E. coli* is under H_2_O_2_ stress [58]. Whether ferrous iron reacts with H2Sn to generate further oxidative stress needs further investigation.

### 3.9 The distribution of OxyR in sequenced bacterial genomes

**Fig. 9:**
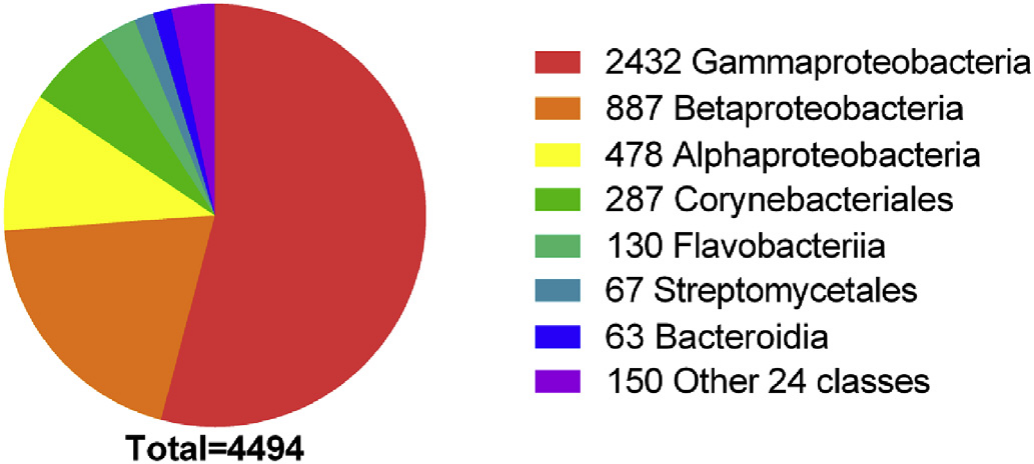
The distribution of OxyRs in sequenced bacterial genomes.

We invested the distribution of OxyR among 8286 microbial genomic sequences (NCBI updated until November 11, 2017) by using BLAST search, and then confirmed with the conserved domain and phylogenetic tree analysis. 4772 identified OxyR distributed in 4494 bacterial genomes, including 2432 Gammaproteobacteria, 887 Bataproteobacteria, 478 Alphaproteobacteria, 287 Corynebacteriales, 130 Flavobacteiriia, 67 Streptomycetales, and 63 Bacterioidia; the other 24 classes had a few genomes containing OxyR (Fig. 9 and Table S1). Thus, OxyR is widely distributed in bacteria, including many obligate anaerobic bacteria in the human gut, such as *Bacteroides* spp., *Prevotella* spp., and *Porphromonas* spp. For anaerobic bacteria, OxyR is likely used to deal with H_2_S_n_ stress.

## 4 Discussion

The levels of cellular sulfane sulfur vary and reach the highest level in early stationary phase for *E. coli* in LB medium (Fig. 1). This observation is shown by using two approaches: the fluorescent probe SSP4 and a constructed reporting system containing a gene regulator inducible by sulfane sulfur. The results are in agreement with previously reported data by using a sulfane sulfur-sensitive green fluorescent protein [59] or by using resonance synchronous spectroscopy [60]. The accumulated sulfane sulfur is rapidly reduced to H_2_S when *E. coli* cells are transferred into fresh LB medium (Fig. 1B), and the reduction is at least in part catalyzed by glutaredoxin and thioredoxin (Fig. 1D). The participation of these enzymes in reducing sulfane sulfur has been reported. Glutaredoxin and thioredoxin are more effective in reducing sulfane sulfur than GSH in *in vitro* assays [61,62], and they can also reduce the level of protein persulfidation *in vivo* [34,35]. Our results support previous reports that these enzymes are involved in reducing sulfane sulfur inside live cells. Further, our results suggest that they play an important role in maintaining cellular sulfane sulfur within a range (Fig. 1D) as well as for the detoxification of added H_2_S_n_.

Several lines of evidence support that OxyR regulates the expression of glutaredoxin and thioredoxin. First, OxyR is known to regulate certain thioredoxin and glutaredoxin; its deletion mutant, containing more sulfane sulfur on average (Fig. 2C), is more sensitive to H_2_S_n_ stress (Fig. 2A&B) than *E. coli wt*. Second, the constructed reporter systems containing the promoters of *trxC, grxA and katG* display OxyR-dependent induction by H_2_S_n_ (Figs. 3 and 4); the two Cys residues C199 and C208 are required for the induction. Third, *in vitro* transcription and translation results show that H_2_S_n_-treated OxyR activates the transcription of *trxC*; again, C199 and C208 are required for the induction (Fig. 5). Fourth, MS analysis confirms the formation of OxyR C199 persulfide (Cys^199^-SSH) upon H_2_S_n_ treatment. The same modification may happen *in vivo* when *E. coli* is confronting H_2_S_n_ stress. C208 is also indispensable in H_2_S_n_ sensing, but its role is unresolved. Although a sulfur bridge between C199 and C208 is possible, our MS data indicated that the sulfur linkage (Cys199-Cys208) is not present in H_2_S_n_-treated OxyR, which is consistent with a previous study indicating that no disulfide bond-linked peptide (Cys199-Cys208) can be identified in H_2_O_2_-treated OxyR [47]. Therefore, OxyR C199 persulfidation is likely the mechanism of sensing H_2_S_n_.

Thus, the H_2_S_n_-treated OxyR activates the expression of glutaredoxin and thioredoxin that reduce H_2_S_n_ to H_2_S in *E. coli* (Graphical abstract). H_2_S_n_-stress also activates the expression of *katG*, and catalase is known to oxidize H_2_S_n_ to sulfur oxides [51] (Graphical abstract).

There are overlaps between the regulated genes under H_2_O_2_ stress or H_2_S_n_ stress (Fig. 8). The difference could result from the modification variations of OxyR by H_2_O_2_ or H_2_S_n_. Three additional modifications on OxyR Cys199 (C199-SNO, C199-SSG and avicinylation) are also known, resulting in different OxyR configurations, DNA binding affinities, and promoter activities [47,63,64]. Therefore, C199-SSH may lead to an allosteric regulation different from the other modifications [47,63,64], acting as one of multi-level transcriptional responses with the other modifications [47]. Further, OxyR is the major gene regulator responding to H_2_O_2_ stress, and other gene regulators can also be affected by H_2_O_2_, including the global gene regulator McbR in *E. coli* [65]. H_2_S_n_ may also affect other gene regulators, contributing to the variations in gene expression under different stresses.

*E. coli* is likely to use house-keeping and induced glutaredoxins and thioredoxins to deal with H_2_S_n_ stress. According to the FPKM (expected number of Fragments Per Kilobase of transcript sequence per millions base pairs sequenced) from the transcriptomic sequencing data, we observed that the basic expression levels of TrxA, TrxB, GrxB, GrxC and GrxD are much higher than those of OxyR-regulated GrxA and TrxC. These proteins are regulated by nutrient mediated regulators and are highly abundant in *E. coli* [[38], [39], [40], [41], [42]], and they may play a “house-keeping” role; whereas, the OxyR activated GrxA, TrxC and KatG are involved in dealing with sulfane sulfur stress. Glutaredoxins and thioredoxins reduce sulfane sulfur to H_2_S, which is released [22,30,37]. For bacteria and animals with sulfide:quinone oxidoreductase, the released H_2_S is captured and oxidized back to sulfane sulfur under aerobic conditions [66]. For *E. coli* and bacteria without sulfide:quinone oxidoreductase, H_2_S will be released and evaporated into the gas phase [19,30]. Under anaerobic conditions, H_2_S is usually released due to the requirement of O_2_ or an alternative electron acceptor for its oxidation [67].

Both S and O are chalcogens. Sulfane sulfur species are similar chemicals to reactive oxygen species (e.g., HSSH *vs* H_2_O_2_) [68], and their modification of proteins is also analogous, i.e., protein-SSH vs protein-SOH [6]. From an evolutionary perspective, the former's history can be traced back before the Great Oxidation Event (GOE), when O_2_ had not been generated by cyanobacteria. As an abundant element on the ancient earth, S should play important roles in ancient microorganisms. Therefore, sulfur metabolism related enzymes should have emerged before the oxygen's era. It is reasonable to speculate that the anti-ROS proteins are derived from anti-sulfane sulfur ones [51]. Our observation that OxyR responses to both reactive oxygen species and sulfane sulfur supports the hypothesis. OxyR is required to deal with H_2_O_2_ only under aerobic conditions; whereas, it responses to H_2_S_n_ under both aerobic and anaerobic conditions. Besides OxyR, the signaling pathway of Keap1/Nrf2 responding to antioxidants is also regulated by polysulfides in mouse neuroblastoma cells [69].

In conclusion, we discovered that *E. coli* uses thioredoxin and glutaredoxin to control homeostasis of intracellular sulfane sulfur. Known bacterial gene regulators sensing sulfane sulfur are specific for activating sulfur-oxidizing genes. OxyR is the first reported global gene factor that functions as a sulfane sulfur sensor via persulfidation of its Cys^199^ under both aerobic and anoxic conditions. This is the fifth type of modification for OxyR activation. Since OxyR is widely distributed in both aerobic and anaerobic bacteria, the OxyR-regulated network may represent a conserved mechanism that bacteria can resort to when confronting endogenous and/or exogenous sulfane sulfur stress.

## Conflicts of interest

No conflict of interests.
