# Supplementary material for: OxyR senses sulfane sulfur and activates the genes for its removal in Escherichia coli
Source: Redox Biol. 2019 Aug 8;26:101293. doi: 10.1016/j.redox.2019.101293 (PMC6831875; doi:10.1016/j.redox.2019.101293)
Supplement: Multimedia component 1 [file mmc1.pdf]

Supplementary Materials

OxyR senses sulfane sulfur and activates the genes for its removal in *Escherichia coli*

Ningke Hou[1], Zhenzhen Yan[1], Kaili Fan[1], Huanjie Li[1], Rui Zhao[1], Yongzhen Xia[1], Luying Xun[1,2#], Huaiwei Liu[1#]

Figure S1-9, Table S1-2

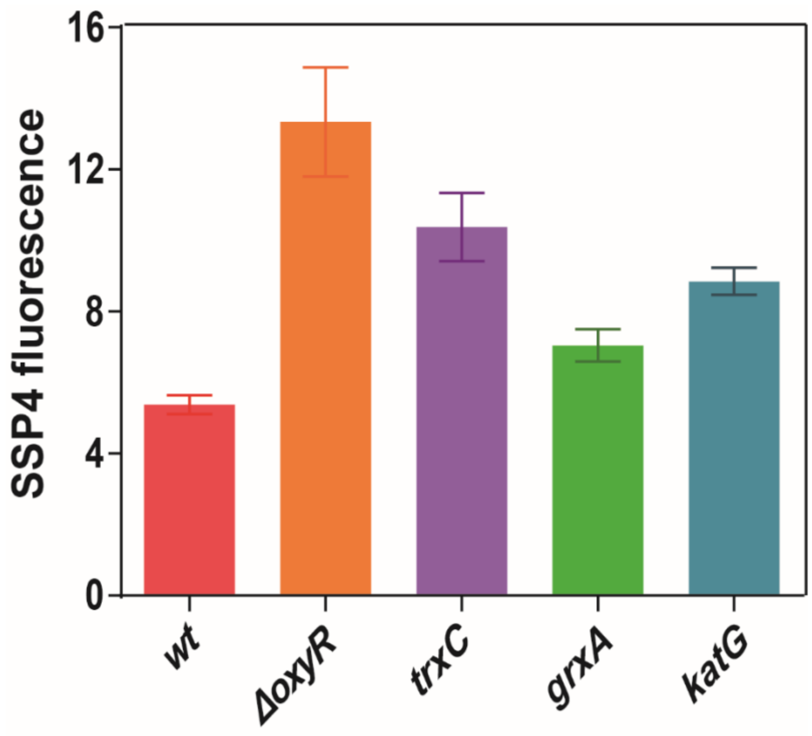

Figure S1. Overexpression of *trxC*, *grxA*, and *katG* in *E. coli ΔoxyR* decreases intracellular sulfane sulfur. *TrxC*, *grxA*, and *katG* genes were expressed with *PlacI* promoter in pTrcHis2A plasmids. Cells were cultured in LB medium until OD_{600} reached 2 and then measured the endogenous sulfane sulfur using SSP4.

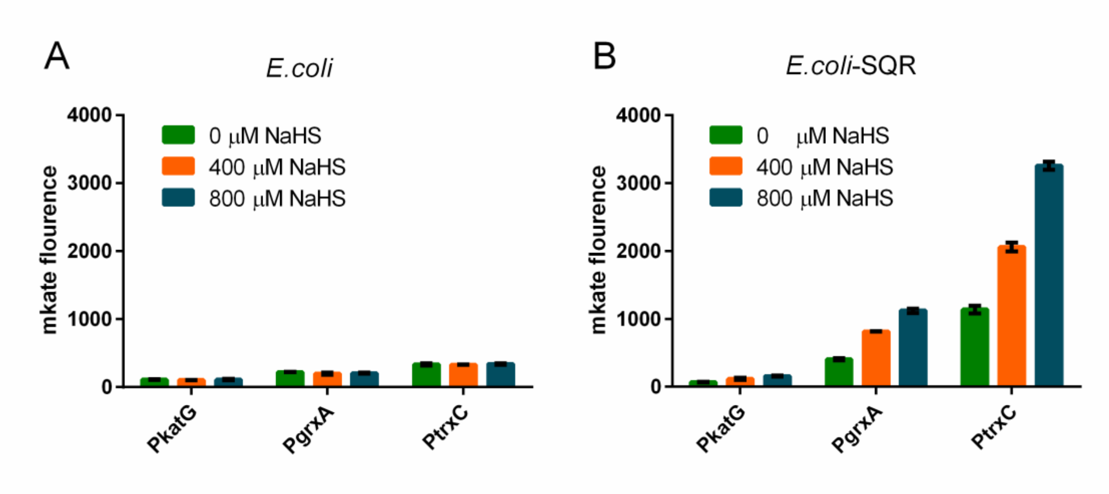

Figure S2. The induction of *trxC, grxA and katG* by NaHS with (A) or without (B) SQR. The *sqr* of *C. pinatubonensis* JMP134 gene was expressed under the P_{lacI} promoter in the pBBrMCS2 plasmid.

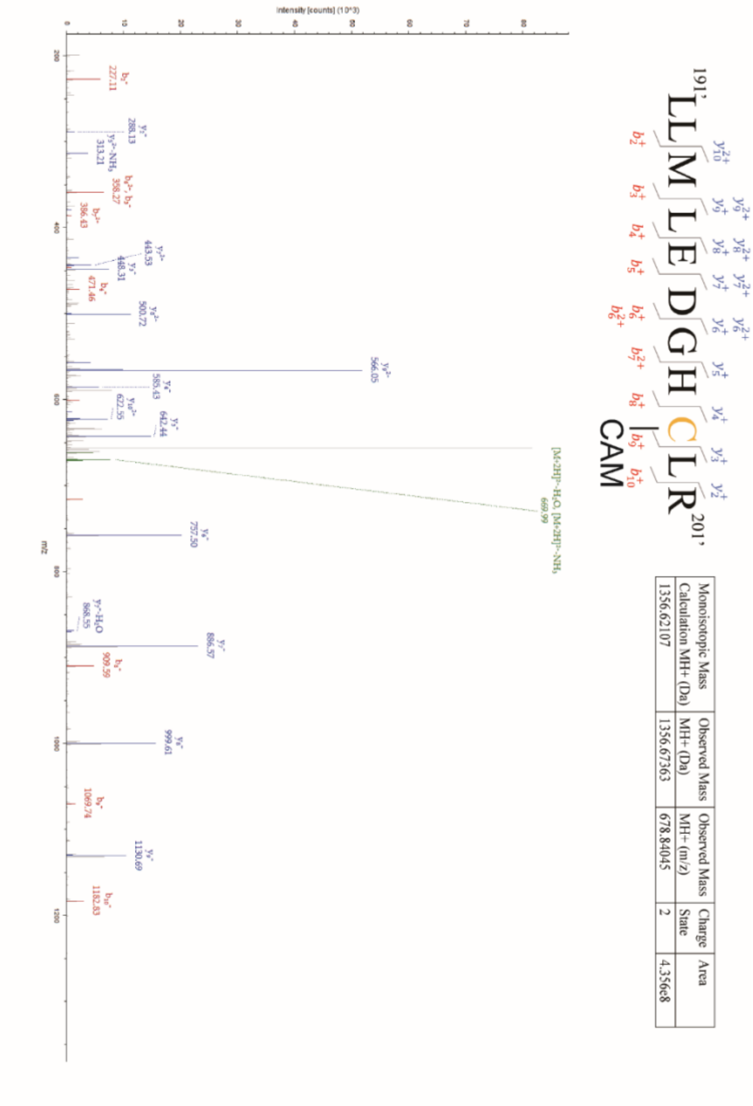

Figure S3. MS[2] data of peptide 1 of H_2S_n-treated OxyR. The sample was digested by trypsin and analyzed by LTQ-Orbitrap Tandem MS. The –SH group was blocked by IAM in the peptide fragment containing Cys_{199}.

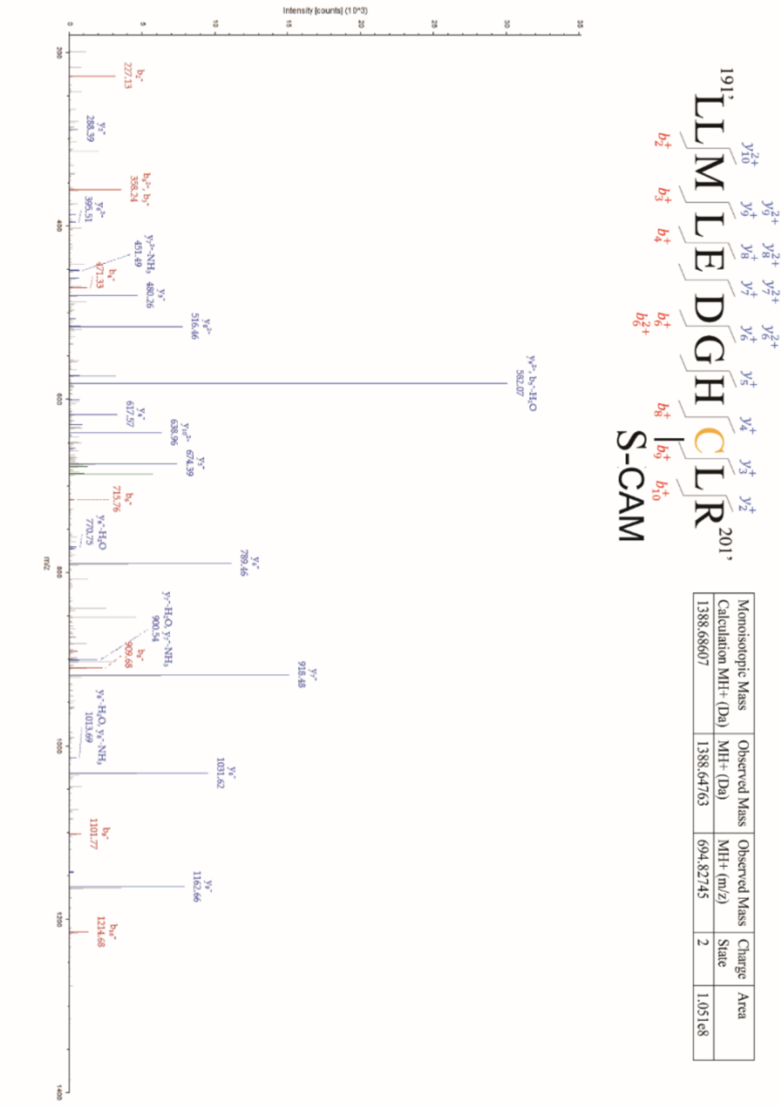

Figure S4. MS[2] data of peptide 2 of H_2S_n-treated OxyR. The sample was digested by trypsin and analyzed by LTQ-Orbitrap Tandem MS. The –SSH group was blocked by IAM in the peptide fragment containing Cys_{199}.

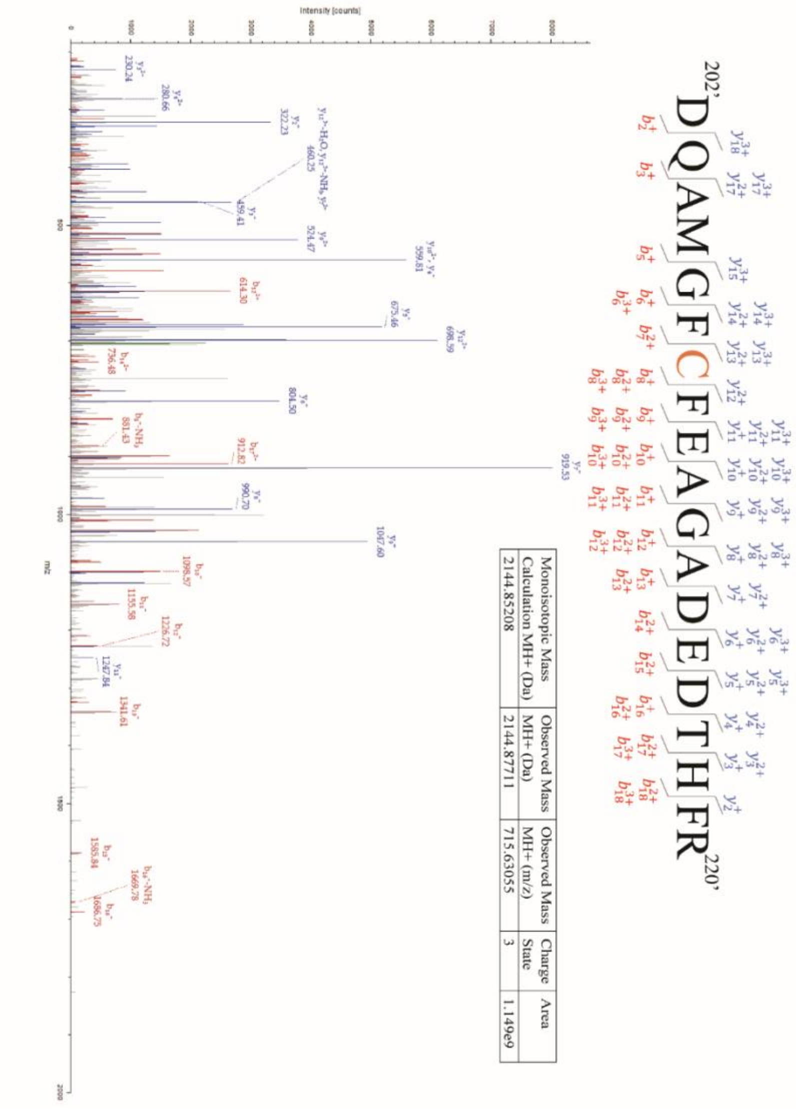

Figure S5. MS² data of peptide 3 of H_2S_n-treated OxyR. The sample was digested by trypsin and analyzed by LTQ-Orbitrap Tandem MS. Cys_{208} contained –SH group in the peptide fragment.

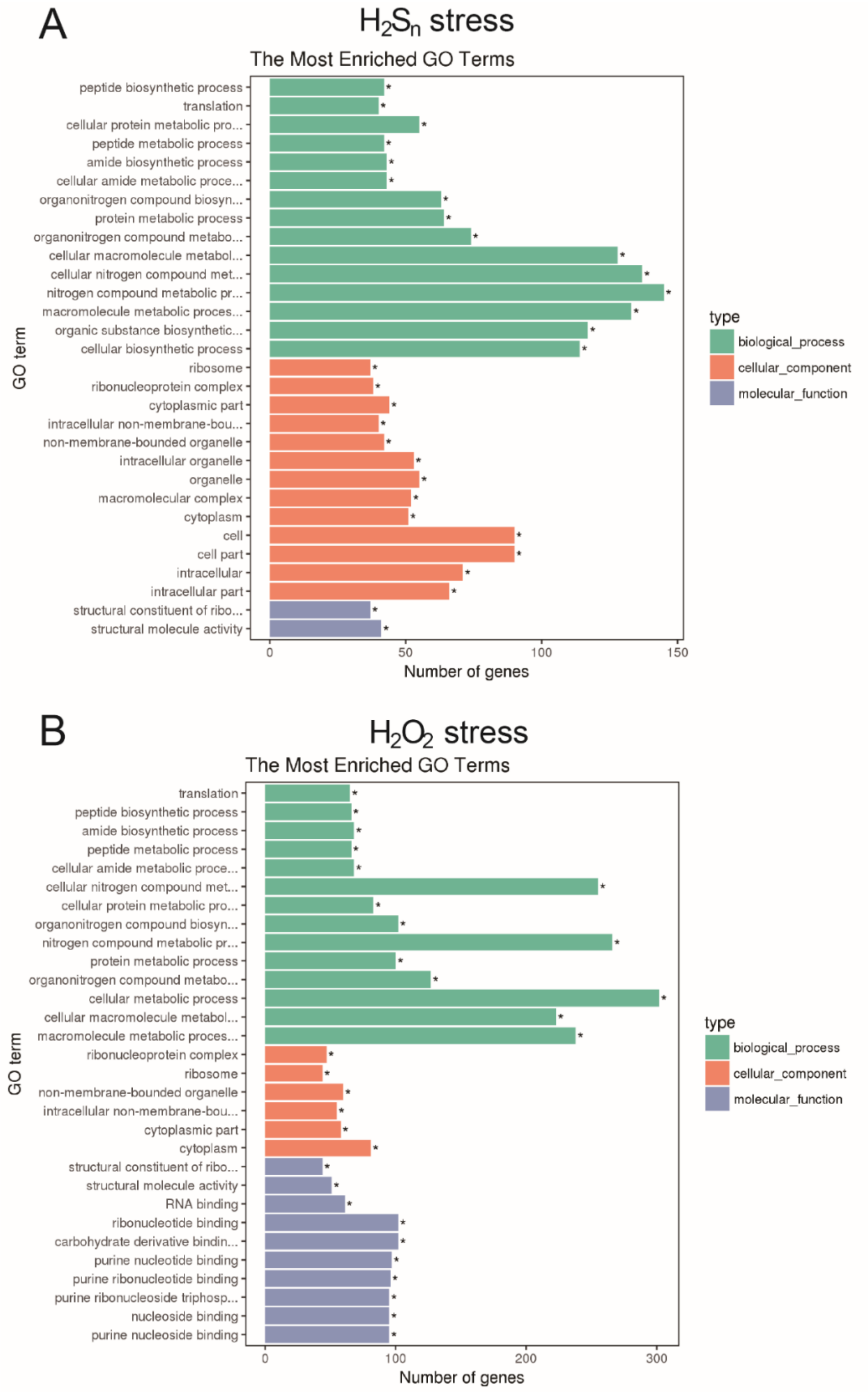

Figure S6. Gene ontology classification of upregulated genes in H_2S_n and H_2O_2 stressed *E. coli*.

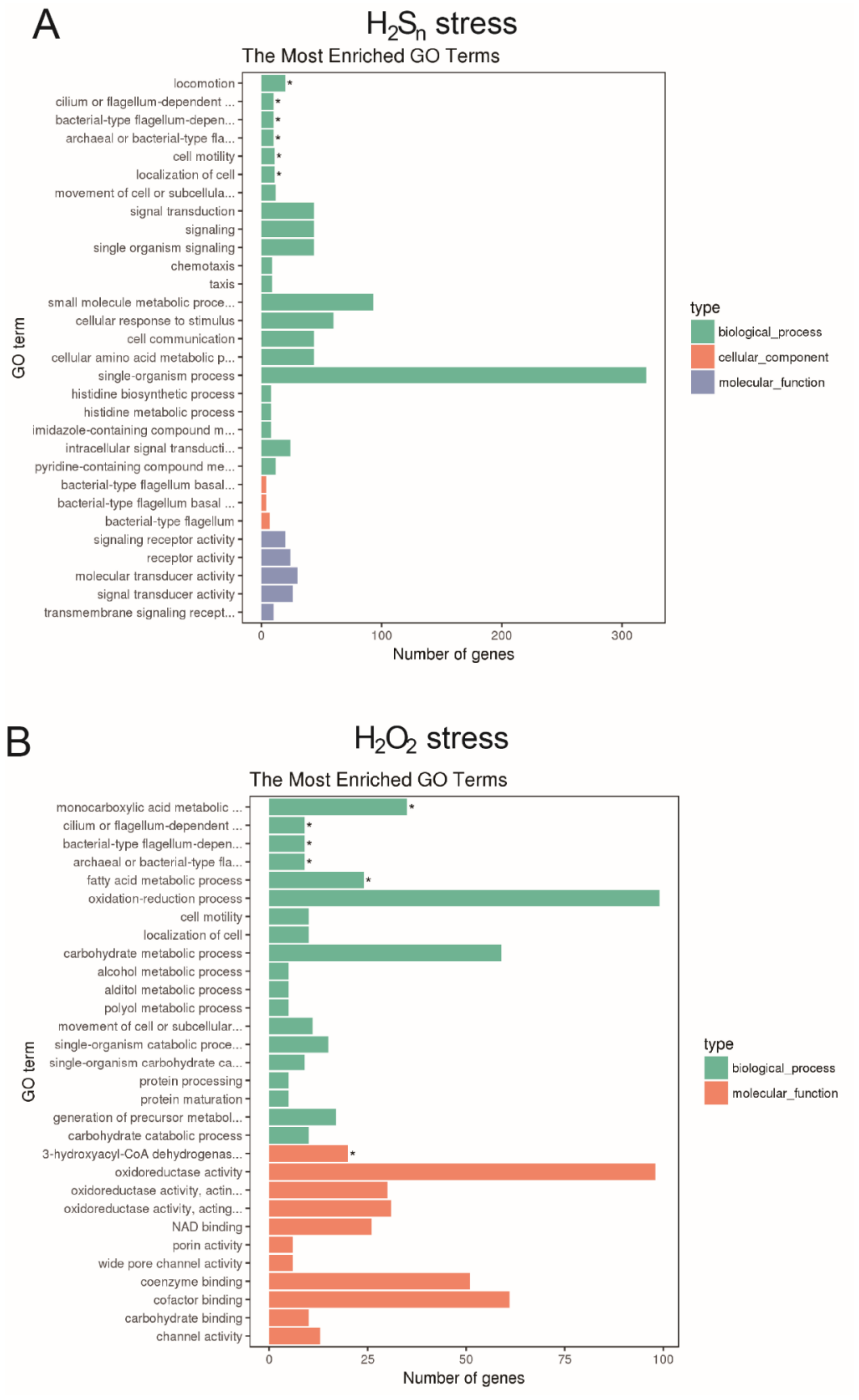

Figure S7. Gene ontology classification of downregulated genes in H_2S_n and H_2O_2 stressed *E. coli*.

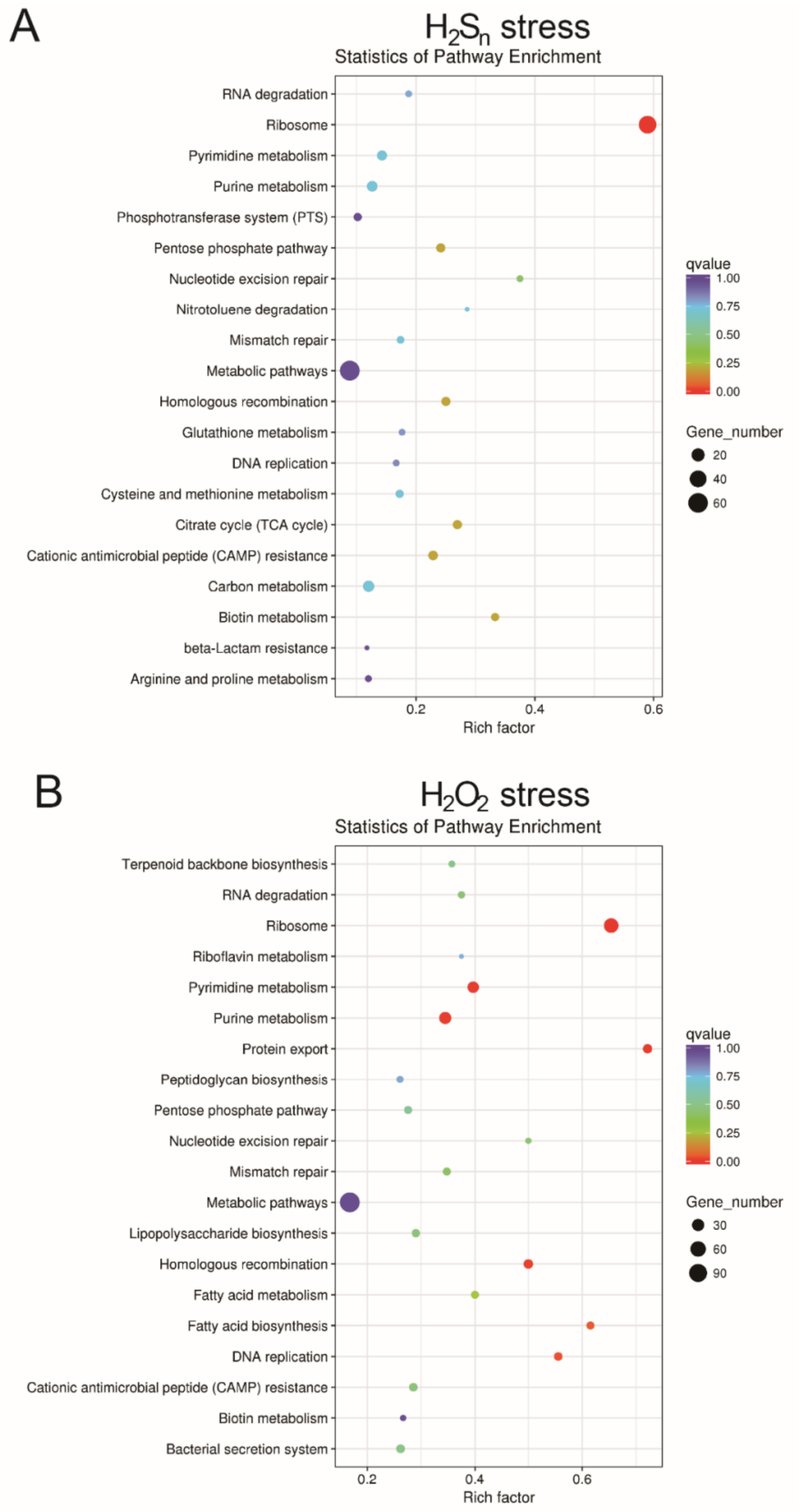

Figure S8. KEGG metabolism pathway classification of upregulated genes in H_2S_n and H_2O_2 stressed *E. coli*.

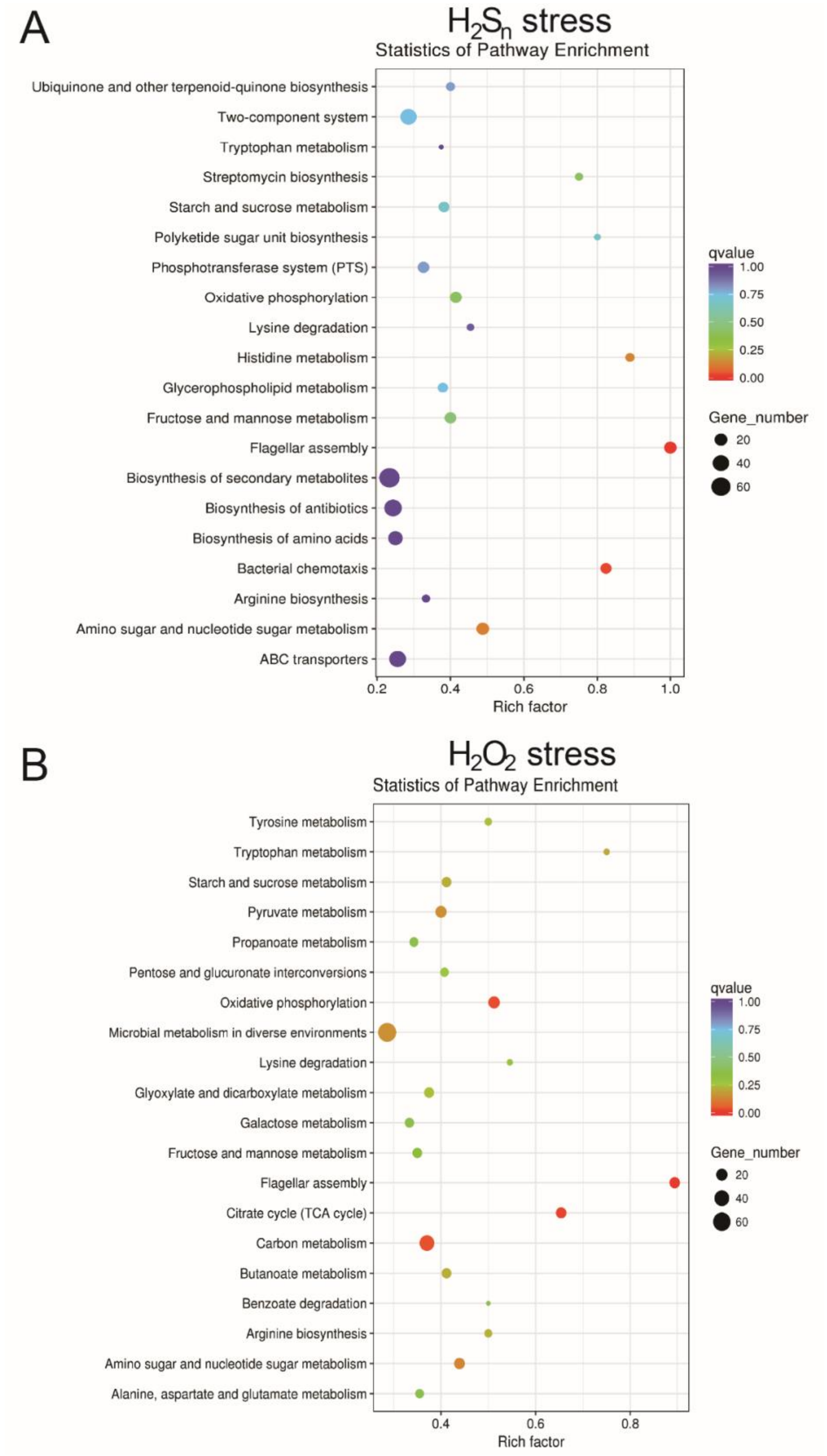

Figure S9. KEGG metabolism pathway classification of downregulated genes in H_2S_n and H_2O_2 stressed *E. coli*.

Table S1. The distribution of OxyRs in sequenced bacterial genomes in class level

Classes name	Counts
Gammaproteobacteria	2432
Betaproteobacteria	887
Alphaproteobacteria	478
Corynebacteriales	287
Flavobacteriia	130
Streptomycetales	67
Bacteroidia	63
Propionibacteriales	26
Cytophagia	24
Deltaproteobacteria	15
Sphingobacteriia	13
Deinococci	11
Oligoflexia	7
Chitinophagia	7
Bifidobacteriales	5
Bacteroidetes Order II. Incertae sedis	5
Planctomycetia	5
Actinomycetales	5
Proteobacteria	4
Nitrospirales	3

Micrococcales	3
Frankiales	3
Saprospiria	2
Spirochaetales	2
Streptosporangiales	2
Leptospirales	2
Ignavibacteria	2
Acidobacteriales	1
Actinobacteria incertae sedis	1
Opitutae	1
Pseudonocardiales	1
**Total**	**4494**

Table S2. Strains and plasmids used in this study

Entry	Strain/plasmid	Characteristic/description
	***Escherichia coli* strains**	
1	DH5α	supE44, AlacU169 (q80lacZAM15), hsdR17, recA1, endA1, gyrA96,thi-1, relA1. For cloning and plasmid construction
2	BL21(DE3)	F-ompT hsdSB (rB-mB-) gal (λ1857 ind1 Sam7 nin5 lacUV5 T7gene1) dcm.
3	BL21(DE3) *ΔoxyR*	BL21(DE3) with *oxyR deletion*
	Plasmids	
4	pTrcHis2A	Amp, Invitrogen.
5	pTrchis2A-P_{katG}-*mkate*-P_{lacI}-*oxyR*	*katG* promoter activity reporter with OxyR
6	pTrchis2A-P_{grxA}-*mkate*-P_{lacI}-*oxyR*	*grxA* promoter activity reporter with OxyR
7	pTrchis2A-P_{trxC}-*mkate*-P_{lacI}-*oxyR*	*trxC* promoter activity reporter with OxyR
8	pTrchis2A-P_{katG}-*mkate*-P_{lacI}-$oxyR_{C199S}$	*katG* promoter activity reporter with $OxyR_{C199S}$
9	pTrchis2A-P_{katG}-*mkate*-P_{lacI}-$oxyR_{C208S}$	*katG* promoter activity reporter with $OxyR_{C208S}$
10	pTrchis2A-P_{katG}-*mkate*-P_{lacI}-$oxyR_{C199S, C208S}$	*katG* promoter activity reporter with $OxyR_{C199S, C208S}$
11	pTrchis2A-P_{grxA}-*mkate*-P_{lacI}-$oxyR_{C199S}$	*grxA* promoter activity reporter with $OxyR_{C199S}$

12	pTrchis2A-*P*$_{grxA}$-*mkate*-*P*$_{lacI}$-*oxyR*$_{\mathrm{C208S}}$	*grxA* promoter activity reporter with OxyR$_{\mathrm{C208S}}$
13	pTrchis2A-*P*$_{grxA}$-*mkate*-*P*$_{lacI}$-*oxyR*$_{\mathrm{C199S,\ C208S}}$	*grxA* promoter activity reporter with OxyR$_{\mathrm{C199S,\ C208S}}$
14	pTrchis2A-*P*$_{trxC}$-*mkate*-*P*$_{lacI}$-*oxyR*$_{\mathrm{C199S}}$	*trxC* promoter activity reporter with OxyR$_{\mathrm{C199S}}$
15	pTrchis2A-*P*$_{trxC}$-*mkate*-*P*$_{lacI}$-*oxyR*$_{\mathrm{C208S}}$	*trxC* promoter activity reporter with OxyR$_{\mathrm{C208S}}$
16	pTrchis2A-*P*$_{trxC}$-*mkate*-*P*$_{lacI}$-*oxyR*$_{\mathrm{C199S,\ C208S}}$	*trxC* promoter activity reporter with OxyR$_{\mathrm{C199S,\ C208S}}$
17	pTrchis2A-*P*$_{katG}$-*mkate*	*katG* promoter activity reporter
18	pTrchis2A-*P*$_{grxA}$-*mkate*	*grxA* promoter activity reporter
19	pTrchis2A-*P*$_{trxC}$-*mkate*	*trxC* promoter activity reporter
20	pCL1920	SPC, low copy plasmid
21	pCL1920-oxyR native promoter-oxyR	For complement *oxyR* to *ΔoxyR* stain
22	pTrchis2A-*P*$_{lacI}$ -*cstR*–*P*$_{op12}$-*mkate*	CstR-based reporter for detecting intracellular polysulfides
23	pTrchis2A-*P*$_{lacI}$-*cstR-mkate-trxA*	CstR-based reporter with *trxA* gene
24	pTrchis2A-*P*$_{lacI}$-*cstR-mkate-trxA-trxB*[a]	CstR-based reporter with *trxA* and *trxB* gene
25	pTrchis2A-*P*$_{lacI}$-*cstR-mkate-grxB*	CstR-based reporter with *grxB* gene
26	pTrchis2A-*P*$_{lacI}$-*cstR-mkate-grxC*	CstR-based reporter with *grxC* gene
27	pTrchis2A-*P*$_{lacI}$-*cstR-mkate-grxD*	CstR-based reporter with *grxD* gene

28	pBBR1mcs2	Kmr, broad host range vector
29	pBBR1mcs2-*katG*	Overexpression of *katG* with *lacI* promoter
30	pBBR1mcs2-*grxA*	Overexpression of *grxA* with *lacI* promoter
31	pBBR1mcs2-*trxC*	Overexpression of *trxC* with *lacI* promoter
32	pET30a	Kmr, expression vector
33	pET30-*oxyR*	Expression and purification of OxyR with a C terminal His-tag
34	pET30-$oxyR_{\mathrm{C199S}}$	Expression and purification of $\mathrm{OxyR}_{\mathrm{C199S}}$ mutant with a C terminal His-tag
35	pET30-$oxyR_{\mathrm{C208S}}$	Expression and purification of $\mathrm{OxyR}_{\mathrm{C208S}}$ mutant with C terminal his-tag
36	pET30-$oxyR_{\mathrm{C199S,\ C208S}}$	Expression and purification of $\mathrm{OxyR}_{\mathrm{C199S,\ C208S}}$ double mutant with C terminal his-tag

[a] An rbs sequence (ggaaggagattaact) was inserted before *trxA* and *trxB*.
